# Community-based rehabilitation workers’ perspectives of wheelchair provision in Uganda: A qualitative study

**DOI:** 10.4102/ajod.v8i0.432

**Published:** 2019-04-24

**Authors:** Nikola Seymour, Martha Geiger, Elsje Scheffler

**Affiliations:** 1 Centre for Rehabilitation Studies, Stellenbosch University, Stellenbosch, South Africa

## Abstract

**Background:**

The challenges of wheelchair provision and use in less resourced settings are the focus of global efforts to enhance wheelchair service delivery. The shortage of professional wheelchair service providers in these settings necessitates the collaboration of multiple stakeholders, including community-based rehabilitation (CBR) workers, whose role needs to be further understood.

**Objectives:**

The aim of this study was to determine what CBR workers in three areas of Uganda perceived as (1) the challenges with wheelchair provision and use, (2) the factors contributing to these challenges, (3) the role they themselves can potentially play and (4) what facilitators they need to achieve this.

**Method:**

This qualitative study in the transformative paradigm comprised focus group discussions to gather perceptions from 21 CBR workers in three areas of Uganda, each with an operational wheelchair service, participant observations and field notes. Thematic analysis of data was implemented.

**Results:**

Community-based rehabilitation workers’ perceptions of challenges were similar while perceived *causes* of challenges differed as influenced by location, historical and current wheelchair availability and the CBR workers’ roles. Their main responsibilities included assistance in overcoming barriers to access the service, transfer of skills and knowledge related to wheelchairs, follow-up of users for wheelchair-related problem-solving, and user and community empowerment.

**Conclusion:**

Community-based rehabilitation workers can contribute in various ways to wheelchair service delivery and inclusion of wheelchair users; however, their capabilities are not consistently applied. Considering the diversity of contextual challenges, CBR workers’ range of responsive approaches, knowledge of networks and ability to work in the community make their input valuable. However, to optimise their contribution, specific planning for their training and financial needs and effective engagement in the wheelchair services delivery system are essential.

**Keywords:**

wheelchairs; less resourced settings; community-based rehabilitation; wheelchair service provision; service steps; Uganda; empowerment; inclusion; assistive device.

## Introduction

The right to personal mobility is mandated by the United Nations Convention on the Rights of Persons with Disability (UNCRPD) (UN 2006). An appropriate wheelchair and related services, information and training are crucial for many persons with disabilities (PWD) and can enhance potential to achieve personal health, development and participation in society (UN 2006; World Health Organization [WHO] 2008). The WHO Guidelines on the Provision of Manual Wheelchairs in Less Resourced Settings recommend that a wheelchair should meet the individual user’s personal and contextual needs and should be provided by suitably trained service providers within a comprehensive service system (WHO 2008).

Wheelchair service delivery includes eight sequential service steps described in Table 1 (WHO 2008). Training of service personnel in the delivery of these steps is required and can have a positive impact on user satisfaction (Borg, Larsson & Östergren 2011a; Toro, Eke & Pearlman 2016; UN 2006; Visagie, Duffield & Unger 2015a). Global resources, such as the WHO Wheelchair Service Training Packages (WSTP), are available to equip service providers and managers in appropriate provision (WHO 2012, 2015).

**TABLE 1:** Service delivery steps.

No.	Step	Description
1	Referral and appointment	Identifying, referring and making appointments for persons with disabilities to ensure equitable access to the wheelchair service.
2	Assessment	Individual assessment to determine needs related to lifestyle, vocation, home environment and physical condition.
3	Prescription	Process to identify wheelchair type and training needs.
4	Funding and ordering	Identifying funding source and ordering the wheelchair.
5	Product preparation	Trained personnel prepare the wheelchair for initial fitting.
6	Fitting	The user tries the wheelchair and final adjustments are carried out to ensure the wheelchair is correctly assembled and set up for the user.
7	User training	Instructions on how to safely and effectively use and maintain the wheelchair.
8	Follow-up, repair and maintenance	Opportunities to check the fit, comfort and stability of the user, ensure the wheelchair is in good working condition and maximise functioning.

*Source*: Adapted from WHO Guidelines. World Health Organization (WHO), 2008, *Guidelines on the provision of manual wheelchairs in less resourced settings*, p. 76, WHO Press, Geneva

The World Disability Report estimates that, in low- and middle-income countries, only 5% – 15% of those needing a wheelchair have what they need (WHO 2011b). While the reasons are multifaceted, these include the lack of appropriately trained personnel (Borg et al. 2011a; Bray et al. 2014; WHO 2011b). Physiotherapists, occupational therapists and orthotists or prosthetists are the most relevant rehabilitation professionals for service delivery, but in many resource-constrained settings they are in short supply (Grut et al. 2012; Mannan, MacLachlan & McAuliffe 2012; Wegner & Rhoda 2015). While WHO recommends other health workers including rehabilitation technicians and community-based rehabilitation (CBR) workers as key stakeholders, scarce training opportunities prevent the appropriate development of skills (WHO 2011a).

Consequently, services are commonly centralised and limited to cities and large towns, impeding access for people from remote and rural communities (WHO 2011a). Furthermore, financial constraints, inaccessible transport and lack of service information together with widespread attitudinal barriers limit the uptake of available services and further disadvantage already marginalised groups (Booyens, Van Pletzen & Lorenzo 2015; Grut et al. 2012; Wegner & Rhoda 2015). Additionally, centralised services may not be responsive to specific contextual barriers, which affect use of the wheelchair, to ensure that the wheelchair can make a real difference (Borg et al. 2011a; Magnusson et al. 2013; Smith, Sakakibara & Miller 2014; Visagie et al. 2015a).

Community-based rehabilitation has been recommended as a strategy to address some of these challenges: stakeholders at the 2006 international consensus conference on wheelchairs pointed out that ‘unless CBR is involved in wheelchair provision, we will not reach very far’ (ISPO 2006:23); indeed, many PWD would not be reached by the available wheelchair services. Community-based rehabilitation, implemented jointly by PWD themselves, community members and service providers, aims to increase inclusion and participation of PWD from a community development and social justice perspective (Nganwa, Batesaki & Mallya 2013; WHO 2010; Wickenden et al. 2012). The CBR approach includes workers or volunteers with a range of titles (here collectively termed CBR workers), providing services and facilitating social inclusion (Booyens et al. 2015; Chappell & Johannsmeier 2009; Deepak et al. 2011; International Labour Organization 2004).

While provision of assistive devices is included under the health domain of the CBR matrix, access to and use of an appropriate device impacts every domain (Heinicke-Motshe 2013; Nganwa et al. 2013; WHO 2010). The combination of CBR workers’ knowledge of local conditions and needs, their availability and accessibility to PWD and their diverse strategies to promote social inclusion together strengthens their potential contribution towards wheelchair service delivery systems and wheelchair use. Identifying and referring those in need of services and encouraging new wheelchair users to participate show how they can be equally valuable to PWD and other wheelchair sector stakeholders (Chappell & Johannsmeier 2009; Deepak et al. 2014; Grut et al. 2012). Also highlighting the potential role of CBR, Borg, Lindstrom and Larsson (2011b) emphasise the need to carry out research and plan the implementation of strategies, which focus on the various components of provision to ensure contextually appropriate, effective and equitable solutions. Despite having an appropriate wheelchair, contextual barriers (such as environmental accessibility, transport, cultural beliefs, negative attitudes and stereotypical assumptions) can further lead to exclusion or discrimination hindering PWD from accessing or using their wheelchairs in a meaningful way (Banda-Chalwe, Nitz & De Jonge 2014; Borg et al. 2012; Smith et al. 2014).

The practice of CBR varies greatly because of its inherent focus on individual needs, the varied organisations initiating CBR programmes and variants of training for CBR workers in different organisations and communities (Chappell & Johannsmeier 2009; Deepak et al. 2011; Wickenden et al. 2012). Nonetheless, a study including 107 CBR workers across seven countries showed that a slight majority of 51% identified ‘technical aids and appliances’ as a major training need (Deepak et al. 2011).

## The situation in Uganda

An overview of key events in the history of wheelchair provision in Uganda is presented in Table 2.

**TABLE 2:** Overview of key events in the wheelchair provision in Uganda.

Year	Historical development	References
1967	The start of the wheelchair sector in Uganda.	Øderud, Brodtkorb & Hotchkiss 2004
1992	CBR adopted by Uganda Ministry of Gender, Labour and Social development (MGLSD).	Abimanyi-Ochom & Mannan 2014
2004	Establishment of The National Wheelchair Coordinating Committee (NWCC). Research in 2005 recommended that a wider range of community organisations pay attention to wheelchair provision.	Mukisa & UNAPD 2005
2006	Launch of Uganda’s National Policy on Disability, inclusive of provision of assistive devices. Highlights the role of civil society organisations and DPOs.	MGLSD 2006:21 & 24
2008	Uganda ratified the UNCRPD.	Abimanyi-Ochom & Mannan 2014
2011	Launch of the ‘Code of practice for design, production, supply and distribution of wheelchairs and tricycles’ drawing on principles of the WHO Wheelchair Guidelines.	UNBS 2015
2013	UNCRPD report by the National Union of Disabled Persons Uganda (NUDIPU) estimated that 80% of PWD are in rural areas. Report indicates that of the total 30% of PWD requiring a mobility device only 2% can access them.	Abimanyi-Ochom & Mannan 2014; NUDIPU 2013
2015	Updated ‘Code of practice for design, production, supply and provision of wheelchairs and tricycles’.	UNBS 2015

UNBS, Uganda National Bureau of Standard; DPO, Disable People’s Organisation; PWD, persons with disabilities; UNCRPD, United Nations Convention on the Rights of Persons with Disability.

Since 1967 when local production of wheelchairs was initiated in Uganda, challenges in the wheelchair sector have included a lack of awareness, insufficient skills and absence of clear roles and responsibilities of stakeholders (Øderud et al. 2004). The establishment of a national wheelchair task force and subsequent launch of the ‘Code of Practice for Design, Production, Supply and Distribution of Wheelchairs and Tricycles’ demonstrate the efforts to address this (Uganda National Bureau of Standard [UNBS] 2015). Updated and relaunched in October 2015, the code of practice lists medical officers, occupational therapists, orthopaedic officers, orthopaedic surgeons, orthopaedic technologists, physiotherapists and wheelchair technologists with appropriate training as potential service providers. Community-based rehabilitation activities are present in Uganda and include the initiation of CBR training through the Community Based Rehabilitation Alliance (COMBRA) in 1994. Community Based Rehabilitation Alliance was involved in developing the wheelchair standards and reference to community involvement in wheelchair service relates to referral and maintenance at community level. Despite these positive steps, substantial gaps remain (Abimanyi-Ochom & Mannan 2014).

## Motivation for this study

Uganda has adopted a CBR approach and the WHO Wheelchair Guidelines (UNBS 2015), and together with international organisations, such as Motivation Charitable Trust, have taken steps towards strengthening the wheelchair provision sector by training service providers and developing services. Feedback from users, CBR workers and service providers indicates both progress and ongoing challenges.

It is crucial to understand the community perspective and include the voice of CBR workers in creating solutions to the complexities and ensuring long-term change (Owusu-Ansah & Mji 2013). The literature specifically exploring the role of CBR workers in wheelchair provision was found to be limited. By exploring this further from the perspectives of CBR workers themselves, the first author, employed by Motivation Charitable Trust, hoped to develop a better understanding of the situation and gather suggestions to improve practice.

## Method

### Research questions

What do CBR workers in three areas of Uganda, each with a wheelchair service, perceive as the challenges with wheelchair provision and use in their communities? How do they think they can assist to overcome these and what facilitators are needed to achieve this?

Objectives were to determine what CBR workers perceive as:

- the challenges with wheelchair provision and use
- the factors contributing to these challenges
- the role they can play
- the facilitators needed to achieve this.

### Study design

A descriptive, qualitative design was applied, with participative aspects as recommended when carrying out research including CBR workers (Deepak et al. 2014; Mannan et al. 2012; Wickenden et al. 2012). Principles of the transformative paradigm, with its philosophical assumptions of addressing social change and starting by gathering community perspectives, enabled space for sharing diverse observations and solutions and created opportunity for learning by both the CBR workers *and* the researcher (Mertens 2007).

### Study setting

**FIGURE 1:**
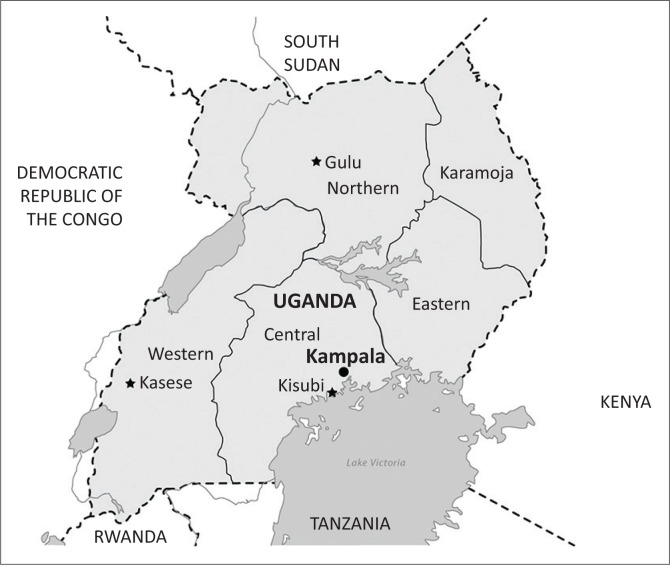
The three areas in Uganda from which participants were recruited: Kisubi in the Central region (bottom right), Kasese in the Eastern region (left) and Gulu in the Northern region (top).

The three areas of Uganda (Figure 1) were purposively chosen, as each had a comprehensive wheelchair service with service steps according to the WHO Wheelchair Guidelines implemented by personnel trained through the WSTP and a CBR programme active in the same target areas as the wheelchair services. The areas were:

- Kisubi, an area both rural and urban, in Wakiso district, 40 km north of the capital, Kampala.
- Kasese, a rural and remote mountainous area in the west.
- Gulu, a predominantly rural area in the north.

The situation in the three areas differed making this a diverse sample. Wheelchair services, all active less than 18 months, were delivered by a non-government hospital, mission hospital and a government district hospital. Personnel from the occupational therapy, physiotherapy and/or orthopaedic technology departments had been trained and were providing the service alongside other professional functions.

The CBR programmes identified per area were operated by three different types of organisations including a department in the same hospital as the wheelchair service, a non-governmental organisation (NGO) working with parents of children with disabilities and disabled people’s organisation. Table 3 summarises the geography and details of the wheelchair services and CBR programmes in each study setting.

**TABLE 3:** Summarised overview of geography, wheelchair services and community-based rehabilitation programmes per study setting.

Variable	1. Central region – Kisubi	2. Western region – Kasese	3. Northern region – Gulu
Description of area	Mix of urban and rural living	Rural, remote & mountainous	Predominantly rural
Wheelchair service delivery by:	NGO rehabilitation hospital and rehabilitation centre	Local mission hospital	District Hospital – Ministry of Health
Description of wheelchair service at time of the study	Active for 1 year 11 months. 3 trained in clinical, technical and management (WSTP and other package). 3 support staff learning on the job	Active for 1 year 3 months. 3 trained in clinical, technical and management (WSTP and other package). 3 support staff learning on the job	Active for 1 year 6 months 6 trained in clinical, technical and management (WSTP and other package). 3 support staff learning on the job
CBR programme delivery by:	Department at the same NGO hospital as the wheelchair service	NGO: Association of parents with children with disabilities	NGO: Disabled Peoples Organisation
Description of activities	CBR programme with workers based at the hospital and network of volunteers in the community	CBR activities with volunteers supporting children and families	Community workers supporting PWD in their homes

CBR, community-based rehabilitation; NGO, non-government organisation; PWD, persons with disabilities.

### Study population, sampling and participants

Purposive sampling was used to identify three community-based organisations (CBOs) who were known by the researcher to be working with the wheelchair services, and then CBR workers working for, or in collaboration with, these CBOs. The inclusion criteria required participants to have a role supporting wheelchair users, to have at least 6 months’ experience working in the particular geographical area and to have worked with a minimum of 10 beneficiaries of the new wheelchair service. Job titles varied, and for the purposes of this article, ‘CBR workers’ was used. To avoid influence of power that researcher or CBO manager bias could have caused, each CBO manager appointed a focal person who, with written information on the purpose of the study, inclusion criteria and ethical considerations, assisted with initial selection and recruitment (Mertens 2007). Focal persons generated a list of up to eight candidates and final selection was agreed jointly with the researcher, on the basis of the inclusion criteria.

The final participant group (Table 4) included one additional person in the west who unexpectedly arrived, out of interest, on the day of data collection. The group in the north was smaller than anticipated because of two ‘no shows’ on the day as a result of challenges with distance and logistics. The final 21 participants across the three focus groups included 11 women, 9 people with disabilities, 2 of whom were wheelchair users. Selections by the focal persons, which were guided by the selection criteria, resulted in participants with a wide range of training and experience, adding to the richness of the data.

**TABLE 4:** Summary of participant profiles.

Variable	Central region – Kisubi	Western region – Kasese	Northern region – Gulu
Total numbers	Total: 7 (6 females + 1 male)	Total: 8 (3 females + 5 males)	Total: 6 (2 females + 4 males)
Participants’ disability status and/or relationship to a wheelchair user	3 PWD (1 wheelchair user)	2 PWD (1 wheelchair user) 4 with a family member using a wheelchair	4 PWD 1 with a family member using a wheelchair
CBR organisation	4 CBR workers based at CBO; 3 volunteers in community (local councillors)	3 CBR workers directly connected with CBO; 4 with other CBOs; 1 based at mission hospital	4 directly connected with CBO; 2 with other CBOs
Professional qualifications; CBR training and experience (in years)	1 occupational therapist; 1 physiotherapist; 1 social worker with additional CBR training. 1 professional CBR qualification. 3 without any training Experience: 3–6 years	5 people had attended the CBR training course delivered through COMBRA Experience: 1–9 years	1 social worker, 1 professional CBR qualification, 1 attended the CBR training course delivered through COMBRA. 4 attended short courses covering aspects of CBR Experience: 1–10 years
Details of past wheelchair training	4 attended 3-day training facilitated by the wheelchair service in 2015. One other had 1 day in 2013	4 had training ranging from 2 h to 2 days in 2014 delivered by the wheelchair service	One person received an orientation

COMBRA, Community-Based Rehabilitation Alliance; CBR, community-based rehabilitation; CBO, community-based organisation; PWD, persons with disabilities.

### Data collection

In each of the three research sites (in the central area, in the west and finally in the north), data collection was done in two steps in March 2015. Thus, all participants first completed a structured self-administered demographic and general information questionnaire in English or in their local language (compiled in Table 3 above). This was followed by a focus group discussion (FGD) led by the researcher and researcher’s assistant. Four key questions aimed at participatory problem identification and solution finding were displayed on a flip chart and posed to the group:

- What are the challenges for people who need or use wheelchairs in your community?
- What are the reasons for these challenges?
- What can you do about it?
- What do you need?

Such an approach is often used in community development (Chambers 1994; 2007; 2010; Freire 1970) and CBR evaluation (Hope & Timmel 1995) and assisted here in creating a picture of the context, including the nuances of multifaceted situations and, moreover, involving participants in identifying their own solutions (Freire 1970; Owusu-Ansah & Mji 2013). The majority of participants contributed in English with others supported by a translator. Prompts were provided by the researcher during the FGDs to ensure all participants had equal opportunity to share. In concluding each FGD, participants were asked what *they* would do with their recommendations, to encourage ownership of the process.

Field notes and the researcher’s reflective journal entries captured observations and thoughts before, during and after each FGD and were also included in analysis, as also described by Birks, Chapman and Francis (2008).

### Data analysis

Data sets from each source, named Area 1 (Central), Area 2 (West) and Area 3 (North), included questionnaire responses translated as needed and transferred to a password protected Excel sheet, FGDs recorded verbatim and the English contributions transcribed, flip charts, field notes and reflective journal entries.

Six phases of thematic analysis as described by Braun and Clarke (2006:87) were applied firstly to each area’s specific focus group and then across areas. Four main themes (reflecting the four questions which in turn reflected the four objectives) were identified: theme one comprised perceived challenges, theme two contributing factors, theme three the possible CBR workers’ role and theme four facilitators to achieve this. The flow of dialogue during the FGDs meant information was not entirely presented in a linear manner according to the four questions but rather interconnected, for example a particular challenge was followed by contributing factors and the CBR role in overcoming it, before moving on to discussing another challenge. Additionally, some challenges were also presented as factors leading to further challenges resulting in one point being coded twice and the decision by the authors to combine theme one and two in the results and findings section of this article. Inductive analysis revealed subthemes for Area 1. Subsequently, analysis of areas 2 and 3 was deductive and, in keeping with flexibility of applied learning in the transformative paradigm, open to new subthemes emerging (Mertens 2007). Coding indicated the area, the theme and the data source, that is, 1 (Area 1)/2 (contributing factor)/FGD11 (page 11 of the FGD transcript).

Once themes and subthemes were identified, the transcriptions were confirmed against the electronically captured flip chart notes and combined with the researcher’s field notes and journal observations into one comprehensive document for each focus group. Finally, a consolidated table was prepared to capture the themes and subthemes from the three data sources (areas 1, 2 and 3) to expose similarities and differences through data triangulation from the three data sources (Carter et al. 2014). Demographic data from the Excel sheet were used to further enrich analysis such as relating their training on wheelchair provision to their responses on training needs.

### Trustworthiness

Qualitative research is by nature idiographic but gathers rich detail of valuable experiences and can enhance learning of complex environments (Carter, Lubinsky & Domholdt 2011:158). No incentives were offered, increasing the possibility that participants had an interest in the topic and were honest with their contributions, especially the groups from west and north, which were held on non-work days. Member checking and a clear audit trail enhanced credibility and transferability, respectively. Although generalising findings to other geographical areas is not the purpose of qualitative research, triangulation of data from the three groups enhanced both credibility and transferability (Mack et al. 2011).

### Ethical considerations

The South African Medical Research Council (MRC) guidelines (MRC 2004) were applied and permissions were obtained from the Stellenbosch University Health Research Ethics Committee (S14/10/210) and the Uganda National Council for Science and Technology. All participants provided written informed consent, either in English or in the applicable regional language or dialect. The researcher was mindful of possible interventionist-researcher bias (O’Leary 2017); so, each organisation and study participants were informed that the study was independent of the researcher’s organisation, and open and honest contributions would enrich the data gathered but no other benefit would be derived from participation.

## Findings and discussion

The findings and the discussion are integrated here to reduce duplication. A tabulated overview of the relationship between the study objectives, the guiding questions, the resultant themes and subthemes, as well as the frameworks used to interpret them is presented in Table 5. Following this, in response to the four study objectives, the four themes will be discussed in pairs, that is, the challenges identified and their contributing factors (themes 1 and 2), followed by the potential roles of CBR workers and facilitators needed to achieve these (themes 3 and 4).

**TABLE 5:** Tabulated overview of the link between the objectives, the guiding questions, frameworks applied (for analysis), themes and subthemes.

Objectives To determine what CBR workers perceive as:	Questions for focus group discussions	Applied frameworks	Themes and subthemes
The challenges with wheelchair provision and use.	‘What are the challenges?’	ICF: Activity limitation and participation restrictions	Theme 1: Challenges identified Mobility limitations Participation restrictions Unavailable and/or unskilled support system Difficulty maintaining health.
The factors contributing to these challenges.	‘What are the reasons for these challenges?’	ICF: Contextual factors: that is, Environmental factors including wheelchairs and 8 wheelchair service steps; personal factors	Theme 2: Contributing factors Inadequate supply of appropriate wheelchairs Inadequate services, systems and policy Attitudes and cultural barriers Inaccessible physical environments Lack of peer role models Poverty.
The role they can play.	‘What can you do about these challenges?’	8 Wheelchair service steps CBR strategies: Empowerment (CBR matrix)	Theme 3: Potential role of CBR workers Facilitate access to services Assist with user training, follow-up, maintenance and repairs Facilitate empowerment and inclusion Gather statistics.
The prerequisite facilitators to achieve this.	‘What do you need in order to do this?’	Training for transformation (Hope & Timmel 1995)	Theme 4: Facilitators needed to achieve this Training related to wheelchairs Communication with wheelchair services Financial resources More CBR workers Recognition of CBR workers in communities and hospitals Opportunities for peer support.

ICF, International Classification of Functioning, Disability and Health; CBR, community-based rehabilitation.

### Themes 1 and 2: Challenges and their contributing factors

In response to objectives 1 and 2, the International Classification of Functioning, Disability and Health (ICF) (WHO 2001) was used to analyse responses contributing to Theme 1 (perceived challenges, including activity limitations and participation restrictions) and Theme 2 (contributing or contextual factors).

#### Mobility Limitations (International Classification of Functioning, Disability and Health: Mobility)

The CBR workers across the three groups indicated that the number of people needing wheelchairs was high. In the west and north, both areas with large rural communities, the need was estimated to be far greater than officially known.

West: ‘Those people who don’t reach into the community think there’s not many disabled persons. Most of these parents hide their children. We as CBR workers know about these people because we’ve been deep in the village.’

Additional reasons for people lacking the necessary mobility device included lack of awareness of the service and policy literacy regarding their rights, low service capacity, lack of appropriate products and attitudinal barriers.

CBR workers from the central and northern areas explained the negative experiences of approaches to providing wheelchairs by some local producers, *ad hoc* political and charity mass distributions and community organisations. Numerous concerns were raised regarding products being provided without individualised service, echoing the damaging effects on health found by Visagie et al. (2015b) and negatively impacting mobility.

Central: ‘Some organisations say I’ve got 50 wheelchairs. Then the issue of not being measured and assessed also comes in, because it’s a gift. Have that one! If it fits you – good! If it doesn’t fit you, you still have it.’

Conversely, for those who did access the new wheelchair services, feedback from the CBR workers highlighted benefits to users, similar to recent findings in Kenya and the Philippines (Williams et al. 2017).

North: ‘There is assessment [ ] they take measurements, [*then*] they make modifications. If they are fitting one chair in the hospital, they are spending a lot of time because they make sure it is modified to fit the child.’

Despite the availability and benefit of the new wheelchair services, the CBR workers identified challenges with access and utilisation. In the north and central areas, apparent gaps between policies and their implementation resulted in confusion for PWD as well as the CBR workers and further reduced use of the available services.

Central: ‘Uganda are supposed to produce wheelchairs, but you are finding because the government doesn’t have a goodwill, there’s no proper funding. The guidelines are also weak and personnel are very few. [ ] That’s why production wheelchairs is very low.’

North: ‘In Uganda the law says the government should assess PWD and provide them with movement facilities. So I think maybe PWD [ ] know their rights, and that’s why they won’t pay.’

North: ‘Our situation is not that we have very few wheelchairs – the wheelchairs are there. Or that the need for the wheelchair is not there – it is there. But they are not given out as fast as possible because people think that it has to go for free.’

Attitudinal barriers were cited as a further reason why people lacked mobility. The groups from the north and west explained that many people were too afraid to access the health facilities in which the new wheelchair services were located fearing negative attitudes and behaviours directed towards them. Persons with disabilities from the north pressured the CBO to continue to provide them with wheelchairs rather than refer them to the hospitals.

North: ‘If one is afraid [*of the hospital*], this means they won’t turn up for the wheelchair even if they are in need.’

This echoes findings in southern Africa where historical and a prevailing medical model approach to disability resulted in fears of PWD regarding discrimination from health providers leading to their avoidance of health institutions (Grut et al. 2012).

According to CBR workers from the north, some people also resisted referrals to the wheelchair service because of past disappointments, which included products promised and not received; services only provided to select groups; or once acquired, the wheelchair not being suitable. Such disappointments result in lack of trust to accept new opportunities (Grut et al. 2012). Furthermore, cultural beliefs played a major role in all areas and prevented carers from wanting the visibility (of the PWD) that a wheelchair affords. It also appeared that people with limited exposure to wheelchairs also had fears regarding negative impacts on the user’s health and functioning, how to use it and of causing damage to it.

Central: ‘I stood on my feet and said no, my child won’t get a wheelchair. That would mean they would never walk again.’

Despite the positive experience of the new service approach, the length of the process and the resulting low output of the services were experienced negatively. Reasons given for the delays and low output in the north included limited service personnel; wheelchair service delivery restricted to 1 day a week; and, as previously highlighted by Bray et al. (2014), the complexity of the type of work. The distances between communities and services also posed challenges to service delivery for both wheelchair service providers and users.

West: ‘Transporting those wheelchairs, [ ] and two technicians from [*the service*] to the outreach is difficult to manage.’

North: ‘You may need to travel to the hospital, maybe twice or even three times to access the chair, and most of the parents give up.’

Earlier findings elsewhere indicate that insufficient maintenance led to premature wear and tear and avoidable break down (Bazant et al. 2017; Toro et al. 2016; Visagie et al. 2015b). Similarly, even where PWD received appropriate, durable products, mobility was impaired over time by the condition of the wheelchair because of parts stolen by community members, high activity levels in rough terrain areas and inappropriate storage. Further reasons surmised include insufficient user skills and knowledge, lack of compliance and difficulty accessing repair services reflecting similar challenges as also reported by Banda-Chalwe et al. (2014).

North: ‘General negligence around maintenance… a simple problem on a wheelchair that could be fixed is usually not done till the problem gets worse.’

West: ‘This repair has to be done in the (service). This parent has no money and the distance is too long.’

#### Participation restrictions (International Classification of Functioning, Disability and Health: Participation restrictions in major life areas and community, social and civic life)

Concurring with findings by Toro et al. (2016) in Indonesia and Borg et al. in Bangladesh (2012), the participants here reported many challenges to participation, even once an appropriate wheelchair was provided, for example:

Central: ‘Sometimes we give wheelchairs to these people, but then it doesn’t change a lot in their quality of life. For example, if a child is school-going, and you give them a wheelchair, but still they stay at home?’

Using a wheelchair in these low income and often rural areas with multiple environmental and attitudinal barriers was reported to result in undue fatigue of the user and family and negatively influence agency. Interestingly, Grut et al. (2012) and Zuurmond et al. (2015) found that this leads to fragmented levels of participation. The daily challenges were made evident in the numerous stories shared.

North: ‘We don’t use the road, we use the path and the path is very narrow. At times we have to cross the river, and there is no bridge, so you have to carry the wheelchair on your back or on a bicycle.’

Central: ‘She stopped over six taxis, but they were all leaving her because she had a wheelchair.’

Central: ‘It’s very expensive for someone who is very poor [ ]. These wheelchairs are bulky. [ ] If you use a Boda (motorbike taxi), then that means you have to get three, one for you, one for the wheelchair and one for your guide.’

West: ‘… their parents regard it as a tiresome exercise – they say they have a lot in terms of looking for survival, and now … getting time to spend on this child…?’

Central: ‘It’s the parents to decide which is more beneficial, him staying with the wheelchair at home, or the wheelchair being kept at school; it can’t be in two places [ ] it means he won’t move [ ], engage in play or interact with peers.’

There was also a lack of wheelchair users as positive role models:

North: ‘Most disabled children that have had limited exposure and mentorship from adult disabled person look at themselves as valueless in the community.’

Lang et al. (2011) earlier warned that few examples of how to be empowered and live a good life may lead to limiting decisions about capability, that is, based on what is thought to be possible rather than what is possible.

#### Unavailable and/or unskilled support system (International Classification of Functioning, Disability and Health: Interpersonal interactions and relationships)

Challenges including those discussed above contributed to high levels of dependence of wheelchair users. Reported caregiver support was however limited by their competing priorities, such as the need to earn an income or care for other children.

Central: ‘If this child has to be wheeled to school, [and] the mother has so many other commitments, he won’t attend school; because she’s the only person to wheel the boy.’

Extended family and community members were in some instances willing to assist, but lack of knowledge and skills impacted on safety and waning interest often reduced reliability.

Central: ‘At first some teachers were willing to do so, but then their attitude changed. I think because he was new [ ] but after he had stayed for a year, it feels like it’s a lot of work for them. Now no-one feels interested to do so.’

However, fears of vulnerability of women and girls also led to rejection of offers of support.

North: ‘Because of such support many especially the females have been objects of sexual abuse. Many because of this will want support from their parents or close relative. Most parents are very protective of the girl child.’

Thus, not participating in activities was at times preferable to requesting support and inconveniencing others.

In many instances, wheelchair users and assistants were reportedly not using the wheelchair correctly or optimally and not taking good care of it. This is similar to findings in other low-resourced settings (Bazant et al. 2017; Toro et al. 2016; Visagie et al. 2015b). Reasons provided in this study included insufficient time spent on training, complexity of the product and product-related information, and general lack of compliance.

West: ‘…parents are trained but on a small scale because of limited time and few service providers and they don’t remember everything.’

Furthermore, the person receiving the initial training from the service was not always the main, only or permanent assistant but rather someone who was available at the time (e.g. the grandmother). Newly learnt skills were often not transferred to others in the family and local community thus further affecting how the wheelchair was used and maintained.

North: ‘The toolbox might be there but there is only a grandmother – don’t even know a spanner – you need someone who has a skill.’

North: ‘Sometimes even the family members are not aware of how to maintain the wheelchair and how to take care for that person. That’s why we find that the wheelchairs get destroyed.’

#### Difficulty maintaining health (International Classification of Functioning, Disability and Health: Self-care)

Wheelchair users struggled to maintain their health because of inappropriate wheelchair designs, misuse of wheelchairs and poorly fitting wheelchairs, factors previously also documented by Scovil et al. (2012). In one example, a child was left in a wheelchair with no one to assist with toileting needs while the parents had to go to the field to work. In another example, a child’s head continuously hung forward because of inadequate wheelchair support. Follow-up services were lacking and yet, according to Bazant et al. (2017) and Visagie et al. (2015b), these could help in identifying unsafe situations and incorrect prescriptions which could lead to further health complications.

Similar to the findings in Zambia (Banda-Chalwe et al. 2014), barriers to physical accessibility led to significant challenges and health concerns.

Central: ‘Kids who are using wheelchairs [ ] have to transfer from a wheelchair and then use their hands and enter in a latrine which is already very dirty. They end up getting secondary infections.’

Furthermore, lack of understanding and insight in the community presented risks to health management (e.g. when school children in the north were disciplined for transferring out of their wheelchairs when they wanted to change positions to relieve pressure). Health is further impacted when health needs are not recognised and medical input is not received timeously.

Central: ‘Because the mum is sick, and the child is not able to wheel herself, she finds herself not going to the hospital, even when she was supposed to get medication.’

### Themes 3 and 4: Possible role of community-based rehabilitation workers and facilitators needed to achieve this

In response to Objective 3, the WHO Wheelchair Service Delivery Steps as well as the WHO (2012) CBR matrix (Empowerment) were used to analyse responses contributing to Theme 3 (the CBR workers’ perceptions of their potential roles). In fulfilment of Objective 4, responses analysed during cross referencing between the three groups contributed to Theme 4 (Facilitators needed to achieve their role).

#### Possible role of community-based rehabilitation workers

The participants here made a range of suggestions for their role. Including typical functions of CBR workers, such as referral, support and empowerment, they also highlighted their potential role in supporting wheelchair service delivery. They illustrated the contextual sensitivity required because of the wide range of challenges. Suggestions showed how their ability to move to the location of the PWD, to the wheelchair service and to other stakeholders and to spend the time needed provided them the opportunity to identify and address some of the diverse challenges. Their experience of working with PWD and understanding of local networks and contextual challenges contributed to various suggestions and further reinforced, as observed by Chappell and Johannsmeier (2009), the importance of the ‘how’ in a CBR worker’s approach. It is not just what CBR workers do but how they do it (e.g. seeking local, contextually appropriate solutions within trusted, community-based relationships that contribute to their effectiveness (Hartley 2004).

The CBR workers who had observed wheelchair service delivery from assessment to fitting and user training commented on the efforts of the service personnel and the positive outcome and suggested that they could assist in the referral process by transferring information and encouraging people to accept referrals.

North: ‘You say that you pay some small amount of money, but the real cost of the wheelchair is almost a million [Uganda Kwacha/USD280]. [ ] If they have understood, then people will start paying that money.’

Other suggestions included arranging for PWD to reach the service by helping to raise funds, source transport and gather groups of PWD together along with facilitating the service providers to plan and prepare for outreach visits. Some of their stories highlighted determination and skills in communication and negotiation as useful traits to be effective – along with resources, such as telephones, airtime or money, to reach people.

West: ‘We talk to [*the users to see if they*] are able to afford the transport that can make [*them*] reach [*the service*]? Then we again talk to the technician. If all are agreed [*then*] we access the service.’

In some instances, accompanying the PWD to the service was perceived as useful to help them locate the service and overcome fears of unfamiliar situations. The impact for the service provider in accurate assessment is inferred in this statement.

West: ‘The CBR worker is known to the parent, [ ] then the parent will [*feel*] at home, then he can be able to elaborate more.’

One participant suggested assisting with product preparation and/or user training during the service to increase service efficiency.

Following up the wheelchair user at home was advocated as a continuum of service for the CBR worked to reinforce, refresh or transfer skills and knowledge regarding use and maintenance of the wheelchair and to assist in overcoming environmental barriers in the home.

North: ‘Those mothers can have enough time with you to ask what they don’t know, and you also have enough time to explain to them and demonstrate.’

West: ‘Caretakers [ ] get tired. So, when they get tired, CBR workers make some follow-ups. You can train another one to carry on with the activity.’

Some participants suggested they could help with maintenance and basic wheelchair repairs during visits and others spoke of the importance of these visits to alert wheelchair services to critical issues needing their input. These follow-up visits would benefit durability and safety of the wheelchair (Chen et al. 2011; Toro et al. 2012). It was evident that the need for follow-up was unpredictable and arose on an *ad hoc* basis, highlighting the value of CBR workers’ involvement as low-resourced wheelchair services can at best offer this on a scheduled basis.

Added benefits of home visits by CBR workers include awareness of and response to a range of common daily difficulties in communities where few people understand wheelchairs (Fefoame, Walugembe & Mpofu 2013). Smith et al. (2014) suggest that wheelchair users faced with complex and multiple challenges would benefit from diverse factors being addressed simultaneously. This was effectively demonstrated in a story from the north in which provision of appropriate wheelchairs for children was accompanied by the CBR worker using his knowledge to inform school management, teachers and pupils to relay fears of disability, advise on accessibility and train school representatives on use of and care for the wheelchair. Such creative and imaginative solutions were also found to overcome barriers elsewhere (Booyens et al. 2015; Lang et al. 2011). Participants from the west recounted how one CBR worker’s physical and caring interaction with a disabled child while feeding her in her wheelchair (purposefully in sight of other community members) challenged their fears and misconceptions that disability is contagious. The impact on some community members was that they in turn challenged others on their fears and the importance of interacting with even the most disabled.

Resilience, determination and resourcefulness along with utilising community networks were shown to impact inclusion elsewhere (Geiser & Boersma 2013; Hansen, Siame & Van der Veen 2014) and here:

North: ‘The community will act as vigilantes to see that assets, wheelchairs for people with disabilities, are protected. If the community is aware they will severely punish whoever causes problems.’

Participants perceived their potential role in identifying and strengthening peer role models and linking wheelchair users to one another for support (Chappell & Johannsmeier 2009). The Accelovate study (Bazant et al. 2017) in Kenya recommended trained peers for supporting PWD, while Booyens et al. (2015) found that CBR workers could draw on empowered PWD to influence leaders and community actors to make change, as was also demonstrated here:

North: ‘If we empower a wheelchair user they will be able to explain their own experience and they [*community leaders*] will listen [ ] CBR role in this is [ ] to connect them with those leaders’.

Some of the CBR workers in the north and central areas demonstrated that their exposure to the range of wheelchairs and provision approaches in their area could benefit wheelchair services, thus confirming earlier evidence (Fefoame et al. 2013). They showed an appreciation of the influence of design and material on durability, safety and function and expressed strong emotions about wheelchair suitability for users. This was despite having received no specific information on the different types and models of wheelchairs available, affecting the quality and efficiency of their feedback.

#### Facilitators needed for community-based rehabilitation workers to achieve their potential role

When the CBR workers were asked what they required to fulfil what they had suggested, all groups identified training, financial resources and collaboration with health services, confirming earlier findings (Booyens et al. 2015; Deepak et al. 2014; Wickenden et al. 2012). The variation in knowledge displayed during the FGD may be expected of CBR workers but also highlights lack of consistent or appropriate knowledge transfer from newly trained wheelchair service providers.

North: ‘What I know is there is a need for capacity building [ ]. We may be doing different things.’

Only 10 of the 21 participants had received training on wheelchairs, and this ranged from 2 h to 3 days. Some recommended that the trainings should be attended by all CBR workers, while another mentioned that parts of the training received were not useful for his role.

Groups suggested similar items for training content including wheelchair types and features; mechanisms to access the service and a better understanding of why a new approach to wheelchair provision is needed. Furthermore, skills in using and maintaining the wheelchair and environmental accessibility and adaptations were also commonly identified as important skills (Heinicke-Motshe 2013; Nganwa et al. 2013). Additional items, such as skills to measure clients, assemble products and carry out basic repairs as well as fundraising, nutrition and early identification, suggested individual or contextual needs. One person reflected how using a wheelchair during a training session increased her understanding and empathy.

Central: ‘It got me thinking this is not really something easy, I really got in their shoes, I must confess it was really hard.’

Close collaboration with the wheelchair services was advocated. For those CBR workers not based at the same location as the wheelchair service, receiving updated information and planning logistics typically depended on the efforts and resources of an individual CBR worker. As suggested by Geiser & Boersma (2013), coordinated mechanisms between CBR workers and wheelchair services would increase efficiency and provide a platform for information transfer, and Borg et al. (2011b) recommend reviewing systems of provision to ensure cost-effectiveness. Two groups also commented on the need to prove their legitimacy and be better recognised in order to enter hospital grounds and communities without challenge. One CBR worker suggested an information pack to clearly show PWD which wheelchairs are available.

Financial resources to make home and service visits possible were mostly lacking with some workers telling how they used their own resources, when available. Resources are thus required, also for empowerment activities such as introducing wheelchair users to peer role models. Some of the workers particularly in the west suggested tools to assist with basic repairs.

Many group contributions displayed passion, commitment and determination similar to that found by Booyens et al. (2015) with community workers in southern Africa. This was despite challenges expressed related to capacity and isolation, especially in the north and west (Fefoame et al. 2013; NUDIPU 2013; Wickenden et al. 2012). The group in the west arrived on a non-work day, some traveling over 2 h without promise of remuneration and stayed engaged for over 3 h. The gathering led them to form a group which continued to meet following the study pointing to their need for peer support.

One CBR worker showed interest in increasing skills to take measurements during follow-up to identify problems and alert the service. As recommended in the Accelovate study (Bazant et al. 2017), it could be useful to train CBR workers to assist with all steps of wheelchair service provision. However, considering the prerequisite facilitators identified to fulfil their role, it would be essential to ensure this was included in a coordinated system and within a broader wheelchair service strategy.

### Limitations

Owing to the limited time frame, human and financial resources in this small scale study for degree purposes, only one FGD was possible per group. Data would have been richer with two or with other data collection methods aiding triangulation (Mack et al. 2011). The findings were analysed manually by the researcher. The use of qualitative data analysis software may have provided a more rigorous analysis.

### Recommendations

The findings indicate that stakeholders interested in developing or improving wheelchair service provision in low income contexts would benefit from engaging with local CBR workers to anticipate the challenges and factors which may affect access to wheelchair services and prevent PWDs from benefitting from an appropriate wheelchair. The potential role of CBR workers and facilitators for this role should be jointly identified with a plan to equip them and ensure effective collaboration. A standardised but flexible training package drawing on the WHO Wheelchair Service Training Packages developed by the international wheelchair or CBR community would simultaneously facilitate consistency and support trainers to adapt it and apply it to their context.

Further engagement with key stakeholders in Uganda could include the findings of the report and explore perspectives of other stakeholders on whether and how to further develop the role of CBR workers in wheelchair service provision. Further research is recommended in areas where CBR workers have been well equipped and purposively engaged to evaluate the impact, broaden the understanding on their role and implement the necessary steps to achieve this.

## Conclusion

The CBR workers in this study identified and described many ongoing challenges for wheelchair users in the areas with wheelchair services, most notably the PWDs’ continued lack of mobility either from not accessing the wheelchair service or because of their wheelchair being damaged or worn out and from limited or inconsistent levels of participation. Perceived reasons were diverse and demonstrated the interaction between contextual barriers prevalent in low income settings with an undeveloped wheelchair service provision system.

With regard to the WHO comprehensive wheelchair service steps, the CBR workers expressed their role in identifying, referring and facilitating access to the service; reinforcing and transferring skills and knowledge in wheelchair use; carrying out home and community visits to follow-up; and contributing to maintain the wheelchair. Further, they can implement strategies for empowering wheelchair users and overcoming environmental barriers to participation. Their inputs on the CBR workers’ potential role indicated their insight to the diverse, observed challenges and highlighted how the attributes of CBR workers could benefit the system. Being at grassroots level, being known to the community and being familiar with the culture and networks equip them to identify issues and navigate solutions. Their commitment to PWD was evident in the wide range of suggestions on how they, with the needed support, could assist.

Determining and formalising the role of the CBR workers in collaboration with the wheelchair service could achieve a degree of consistency in their role, enable comparability and ensure that the wheelchair service can benefit from their grassroots experience. Suggestions to achieve and maintain this included provision of training and financial resources and establishing effective communications between the CBR workers and the wheelchair service providers.
